# Identification of novel sarcoma risk genes using a two-stage genome wide DNA sequencing strategy in cancer cluster families and population case and control cohorts

**DOI:** 10.1186/s12881-019-0808-9

**Published:** 2019-05-03

**Authors:** Rachel M. Jones, Phillip E. Melton, Mark Pinese, Alexander J. Rea, Evan Ingley, Mandy L. Ballinger, International Sarcoma Kindred Study, David J. Wood, David M. Thomas, Eric K. Moses

**Affiliations:** 1 The Curtin UWA Centre for Genetic Origins of Health and Disease, Faculty of Health Sciences, Curtin University and Faculty of Health and Medical Sciences, M409 The University of Western Australia, 35 Stirling Hwy, Crawley, 6009 Western Australia; 2 Medical School, Faculty of Health and Medical Sciences, University of Western Australia, Crawley, Australia; 3 School of Pharmacy and Biomedical Sciences, Faculty of Health Sciences, Curtin University, Bentley, Western Australia; 4 Cancer Division, Garvan Institute of Medical Research, Darlinghurst, NSW Australia; 5 School of Veterinary and Life Sciences, Murdoch University, Murdoch, Australia; 6 Harry Perkins Institute of Medical Research, Murdoch, Western Australia; 7 The Centre for Medical Research, The University of Western Australia, Crawley, Australia; 8 School of Biomedical Sciences, Faculty of Health and Medical Sciences, The University of Western Australia, Crawley, Australia

**Keywords:** Sarcoma, Whole exome sequencing, Cancer cluster families, Genetic risk variants, Whole genome sequencing

## Abstract

**Background:**

Although familial clustering of cancers is relatively common, only a small proportion of familial cancer risk can be explained by known cancer predisposition genes.

**Methods:**

In this study we employed a two-stage approach to identify candidate sarcoma risk genes. First, we conducted whole exome sequencing in three multigenerational cancer families ascertained through a sarcoma proband (*n* = 19) in order to prioritize candidate genes for validation in an independent case-control cohort of sarcoma patients using family-based association and segregation analysis. The second stage employed a burden analysis of rare variants within prioritized candidate genes identified from stage one in 560 sarcoma cases and 1144 healthy ageing controls, for which whole genome sequence was available.

**Results:**

Variants from eight genes were identified in stage one. Following gene-based burden testing and after correction for multiple testing, two of these genes, *ABCB5* and *C16orf96*, were determined to show statistically significant association with cancer. The *ABCB5* gene was found to have a higher burden of putative regulatory variants (OR = 4.9*, p*-value = 0.007, q-value = 0.04) based on allele counts in sarcoma cases compared to controls. *C16orf96,* was found to have a significantly lower burden (OR = 0.58*, p*-value = 0.0004, q-value = 0.003) of regulatory variants in controls compared to sarcoma cases.

**Conclusions:**

Based on these genetic association data we propose that *ABCB5* and *C16orf96* are novel candidate risk genes for sarcoma. Although neither of these two genes have been previously associated with sarcoma, *ABCB5* has been shown to share clinical drug resistance associations with melanoma and leukaemia and *C16orf96* shares regulatory elements with genes that are involved with TNF-alpha mediated apoptosis in a p53/TP53-dependent manner. Future genetic studies in other family and population cohorts will be required for further validation of these novel findings.

**Electronic supplementary material:**

The online version of this article (10.1186/s12881-019-0808-9) contains supplementary material, which is available to authorized users.

## Background

Cancers are a major cause of morbidity and mortality in the world today. Cancers can be caused by mutations that arise in single somatic cells resulting in sporadic tumors, or by heritable germline susceptibility variants [1]. Familial clustering of cancers is relatively common [2]. Although more than 100 cancer susceptibility genes have been identified using a variety of genetic strategies [3–6], a large proportion of familial risk remains to be accounted for [7, 8]. The study of cancer families using contemporary genome-wide DNA sequencing technologies now offers an opportunity to identify novel germline risk variants and potentially novel gene targets that will be of clinical utility for better prediction of cancer risk and improved therapeutic intervention. For example, gene variants that regulate drug metabolism can influence response to treatment and are of interest as a target for improved therapeutic intervention [9].

Recently there has been a return to family-based study designs to identify rare risk variants involved in complex human disease and traits, with the underlying assumption that affected members of the same family will carry the same rare risk variant [10–14]. In a family-based study design, the number of individuals needed for rare variant discovery is potentially fewer than in population cohorts of unrelated individuals [10]. Two-stage next generation sequencing family study designs are recommended. In the first stage, family members are sequenced and identified variants are ranked according to their likelihood of being associated with the disease or trait [15]. In the second stage, variants are tested for disease association in an independent population-based sample [15].

Sarcomas are a rare group of cancers that arise predominantly in the connective tissues of the body [16]. Despite representing only 1% of all cancers, sarcomas are a high impact group of cancers that disproportionately affect children, adolescents and young adults [17]. Families in which related individuals develop a rare form of cancer, such as sarcoma, are more likely to have a heritable susceptibility variant segregating in a cancer risk gene compared to families affected by more common types of cancer [18]. In this study, we have used whole exome sequencing (WES) of germline DNA to identify novel candidate sarcoma risk genes in three multigenerational mixed cancer pedigrees identified by a sarcoma proband from the International Sarcoma Kindred Study (ISKS) [19]. The identified candidate risk genes were validated by variant burden analyses using whole genome sequencing data from sarcoma cases from the ISKS and healthy ageing controls from the Medical Genome Reference Bank (MGRB) [20].

## Methods

### Initial discovery cohort

#### Samples for whole exome sequencing

**Fig. 1:**
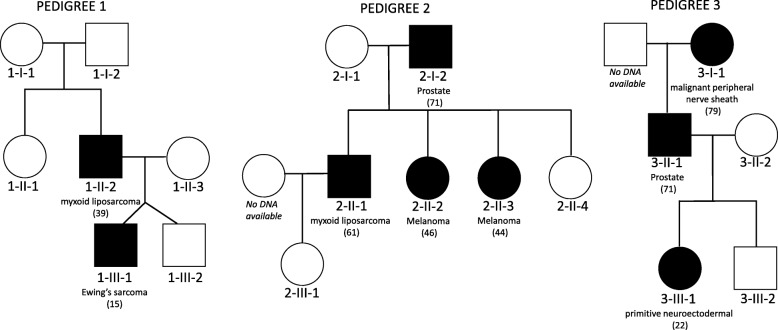
Pedigrees of three cancer cluster pedigrees identified by a sarcoma proband

An initial discovery cohort of three cancer cluster pedigrees (Fig. 1) with a sarcoma proband were selected from the ISKS. The ISKS is a global genetic, biological, epidemiological, and clinical resource available for researchers to investigate hereditary characteristics of sarcoma (see Additional file 1) [19, 21].

In selecting this initial discovery cohort, sarcoma pedigrees were chosen that are not defined by or associated with 72 known cancer susceptibility gene as previously reported [19] and that have at least one first degree relative of the sarcoma proband with a cancer diagnosis and at least one unaffected relative with germline DNA available for WES. Pedigree 1 (Fig. 1) includes a proband (Patient 1-III-1) who developed Ewing’s sarcoma at 15 years of age, as well as a non-identical twin brother (Patient 1-III-2) who has not developed sarcoma. The proband’s father (Patient 1-II-2) developed myxoid liposarcoma at 39 years of age. Germline DNA was available from the proband and father, and from the proband’s twin brother, mother (Patient 1-II-3), an aunt (Patient 1-II-1) and grandparents (Patient 1-I-1 and Patient 1-I-2), who were all unaffected by cancer.

Pedigree 2 (Fig. 1) was identified by a proband (Patient 2-II-1) who developed myxoid liposarcoma at 61 years of age. The proband’s father (Patient 2-I-2) developed prostate cancer at 71 years old, and two of the proband’s sisters were diagnosed with skin melanomas at 44 (Patient 2-II-3) and 46 (Patient 2-II-2) years of age. Germline DNA was available for the proband, one of his unaffected children (Patient 2-III-1), three of his sisters (including an unaffected sister, Patient 2-II-4), and his parents (Patient 2-I-1 and Patient 2–1-2).

In Pedigree 3 (Fig. 1), there are two individuals with sarcoma: the proband (Patient 3-III-1) who developed a primitive neuroectodermal tumour at 22 years of age, and her grandmother (Patient 3-I-1) who developed malignant peripheral nerve sheath tumour at 79 years old. The proband’s father (Patient 3-II-1) was diagnosed with prostate cancer at 51 years of age, and the proband’s aunt developed breast cancer at age 36. Germline DNA was available from the proband, her parents (Patient 3-II-1 and Patient 3-II-2), her unaffected brother (Patient 3-III-2), and her grandmother (Patient 3-I-1).

#### Whole exome sequencing

Nineteen individuals (9 cancer cases and 10 unaffected family members) from these three ISKS family pedigrees underwent germline WES. Anti-coagulated blood was processed using a Ficoll gradient and DNA was extracted from the nucleated cell product using QIAamp DNA blood kit (Qiagen, Germany). Whole genome amplification was performed on two of these germline DNA samples (Patient 3-I-1 and Patient 3-III-2) that were badly degraded, using a Qiagen REPLI-g Mini Kit as per the manufacturer’s instructions. Exome library preparation was performed using the Thermo Fisher Scientific Ion AmpliSeq™ Exome RDY Kit. The target regions were amplified using the Ion Ampliseq™ Exome RDY Library Preparation. Validation of enrichment and quantification of target DNA were performed on the ViiA 7 (Thermo Fisher Scientific). Libraries were loaded onto the Ion P1 v2 BC chip (Thermo Fisher Scientific) using the Ion Chef™ and sequenced on the Ion Proton™ as per the manufacturer’s instructions.

#### Sequence alignment and variant calling

Base calling was performed using the Torrent Variant Caller (Life Technologies, version 5.0.0) using the AmpliSeq Exome capture .bed file. Each of the 19 participants was called individually and then merged using BCFtools [22] *vcf-merge* to create a single *.vcf file. BCFtools [22] *missing-to-reference* was also run on the merged file to fill unknown positions to homozygous reference (0/0).

Genome Analysis Toolkit [23] *UnifiedGenotyper* (version 3.4.0) was used in addition to the single sample calling to sort, index and call the *.bam files to ensure base calling accuracy.

**Fig. 2:**
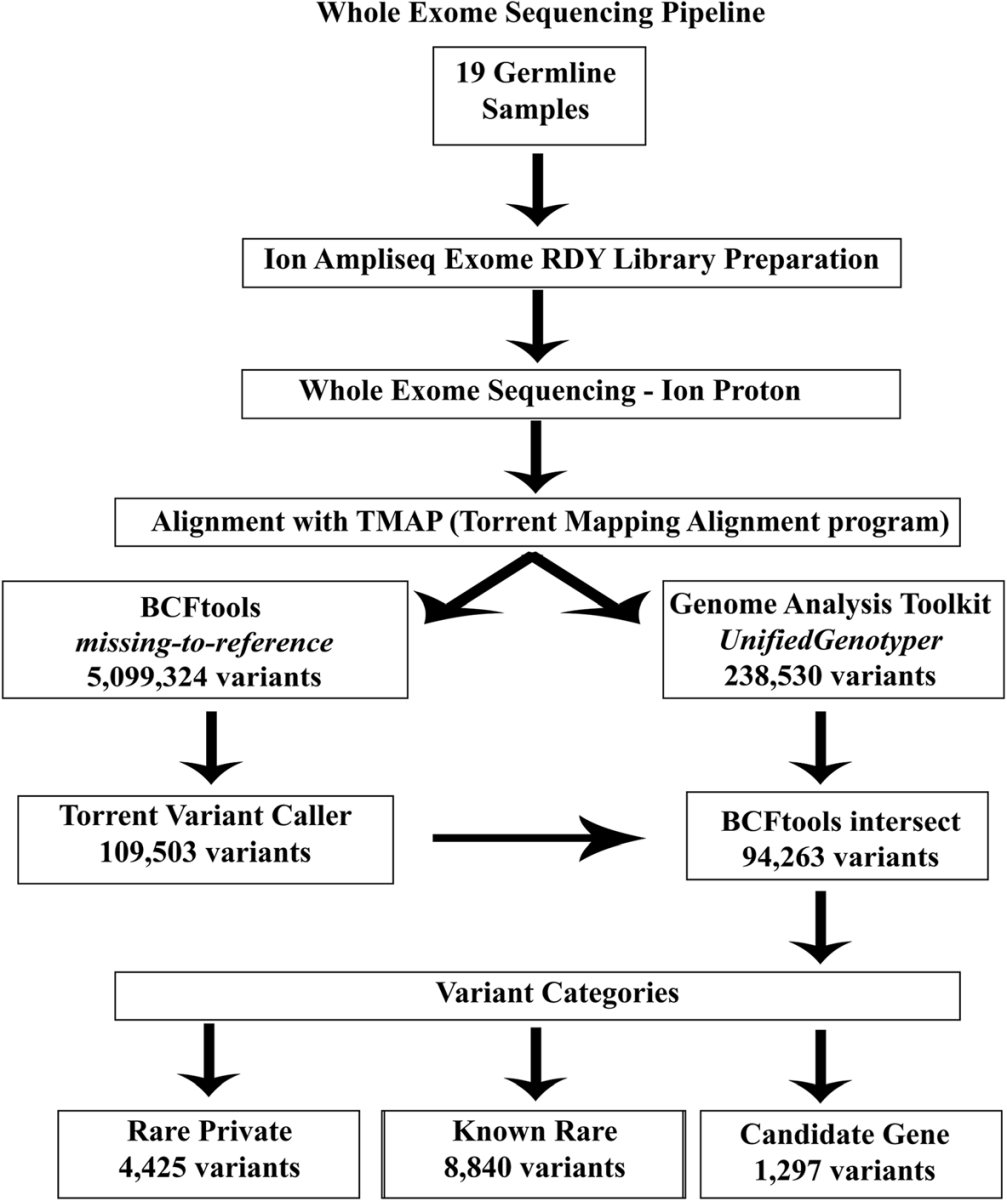
Bioinformatics flowchart for variant calling in pedigree samples

The resulting *.vcf files from both Torrent Variant Caller and Genome Analysis Toolkit [23] were combined using BCFtools [22] intersect (*isec*) exact allele match to identify the common calls between these two bioinformatics tools. The intersect data from both callers was used for the remainder of the analysis to improve confidence that base calls were real and not sequence artefact. Details of this pipeline are shown in Fig. 2.

#### Variant categorization

Three different categories of variants were selected for further analyses. The first category were rare private variants; defined as those unique to individuals or pedigrees [24]. To identify rare private variants in this study, the variants from the intersect file were filtered to remove variants with an rs ID number (build hg19).

The second category were known rare variants. Variants that were present in both the intersect file and the full Exome Aggregation Consortium browser (version 0.3.1, downloaded 30 August 2016) with a minor allele frequency ≤ 0.01 (1%) were selected.

The third category were variants within candidate genes that were selected based on a priori knowledge of cancer biology. Variants in 118 known cancer and sarcoma genes, and in genes 25 kb upstream and downstream of each known gene to include any potential regulatory variants captured in off-target reads, were identified from the intersect file (see Additional file 1: Table S1). The 118 known cancer and sarcoma genes were selected from the HaloPlex Cancer Research Panel, Illumina’s MiSeq and TruSeq Cancer Panels, and the Online Mendelian Inheritance in Man database [6].

#### Association analysis

A weighted covariate using a probability unit (probit) regression was created in R (bias reduction in binomial-response generalized linear models library, version 3.1.2) to account for ascertainment bias. Probit regression assigns a weight to each individual based on their case status and can be used as a covariate in modelling.

Sequential Oligogenic Linkage Analysis Routines (SOLAR) was employed to estimate and test the significance of association under a polygenic statistical model for four cancer outcomes. These four cancer outcomes were 1) age at onset of cancer; 2) age at onset of sarcoma; 3) any cancer; and 4) any sarcoma [25]. Covariates included age and sex, and their interactions along with a weighting factor were assigned to each individual to correct for ascertainment bias. Analysis of any cancer or any sarcoma treated as discrete binary traits was performed using a liability threshold model. This model employs probit regression for the mean effect component and a standard random effects variance component model for the residual additive genetic component of variance. As variance component models are highly influenced by kurtosis, the age of cancer onset and age of sarcoma onset were inverse normalized using the *inorm* function in SOLAR.

#### Bonferroni correction

Bonferroni correction was performed on each annotated variant list to correct for multiple testing. Corrections were performed for each method based on the number of variants in the prioritized list. Any significant variants after correcting for multiple testing, or nominal variants (*p*-value < 0.05), were investigated for co-segregation in the pedigrees.

#### Segregation analysis

Three assumptions were used to determine familial segregation. First, the variant will be rare (shared only by cases in one family pedigree). Second, every carrier of a putative disease-causing variant will have the phenotype (complete penetrance). Third, every individual with the disorder will carry the putative disease-causing variant (100% probability of observing a genotype given the phenotype).

### Gene validation

#### Replication cohort

Variant burden analyses were performed for candidate risk genes using whole genome sequencing data from an additional 560 sarcoma cases from the ISKS and 1144 healthy ageing cancer-free controls selected from the MGRB program [20, 26] accounting for European ancestry. The MGRB program is described further in the Additional file 1.

#### Whole genome sequencing

Whole genome sequencing for ISKS cases and MGRB controls was performed at one lane per sample on the Illumina HiSeq X Ten platform using TruSeq Nano chemistry (2 × 150 base pair paired-end reads, > 30X mean depth for all samples). Samples passing FastQC390 and *verifyBamID* contamination filters were mapped to the 1000 Genomes Project hs37d5 reference with additional PhiX decoy, and small variants called using the Genome Analysis Toolkit 3.7 best practices pipeline. Variants passing variant quality score recalibration tranche thresholds of 99.5% (single nucleotide polymorphisms) and 99.0% (insertions and deletions) were retained for frequency summarization.

#### Annotation of variants in candidate genes

Variant calling files for genes identified in the family-based analyses were obtained from the WGS dataset and annotated using ANNOVAR (version 2015Jun16) and RegulomeDB [27, 28].

#### Variant annotation and filtering

ANNOVAR [29] was used to annotate the intersect file using gene-based annotation. Variant filtering retained loci if they were: stop-gain or stop-loss, predicted to be deleterious or probably damaging in SIFT (Sorting Intolerant from Tolerant) [30] and PolyPhen-2 [31] and had a Genomic Evolutionary Rate Profiling [32] score < 3. All remaining variants were annotated using RegulomeDB [28]. Variants that had a RegulomeDB score < 3 were retained as these scores represent the highest confidence that a variant lies within a regulatory region.

#### Variant burden analyses

The total number of rare (minor allele frequency (MAF < 0.05)) nonsynonymous and deleterious alleles (defined as deleterious in both SIFT and PolyPhen-2) and the total number of major alleles in each candidate gene of interest were determined and compared between ISKS cases and MGRB controls. Similarly, the total number of rare (MAF < 0.05) putative regulatory alleles (defined as those with a RegulomeDB score < 3) and the total number of major alleles in each candidate gene of interest were determined and compared between ISKS cases and MGRB controls.

Odds ratios and *p*-values reported for variant burden analysis were obtained from one-sided Fisher’s exact tests performed in R to compare total burden of deleterious and putative regulatory variants, separately, in ISKS cases and MGRB controls. A conservative Bonferroni approach was used to correct for multiple testing.

## Results

### Discovery of candidate risk variants

Three multigenerational mixed cancer cluster pedigrees (9 cancer cases and 10 family members) were selected from the ISKS for variant discovery (Fig. 1). The average age of onset of cancers in the three pedigrees is similar to the average age of onset of all cancers and age of onset of sarcomas in the ISKS (Table 1).

**Table 1:** Study cohort demographics [19]

Variable	Study	ISKS^a^
Sex	11 female (58%)	–
Average age of patients	55.3 years (range: 15–90 years)	–
Average age of cancer onset	47.5 years (range: 15–79 years)	47 years (range: 1 month – 93 years)
Average age of sarcoma onset	–	46 years (range: 3–93 years)

^a^*ISKS* International Sarcoma Kindred Cohort

#### Whole exome sequencing

The average depth of coverage across all samples was 100X (range 72-131X). The average number of reads, mapped to hg19, was 38,484,361 and the average total genotyping rate was 98.9%.

Variants called from both Torrent Variant Caller and Genome Analysis Toolkit were intersected using the bioinformatic software, BCFtools in order to improve confidence that base calls were real and not sequence artefact. In total, 109,503 variants were called by Torrent Variant Caller and 238,530 variants were called by Genome Analysis Toolkit *UnifiedGenotyper*. The intersect file from both callers contained 94,263 variants for all 19 subjects (Fig. 2).

#### Variant annotation and filtering

The intersected variant calling file was annotated using Annotate Variation (ANNOVAR) [29] and Regulome Database (RegulomeDB) [28] and the annotations were used to filter putative nonsynonymous and regulatory variants. Approximately 42% of variants were exonic and 51% were intronic. Less than 1% of variants were intergenic. Of the exonic variants, approximately 48% were nonsynonymous and 51% were synonymous, with 0.5% classified as stop gain and loss variants (Table 2).

**Table 2:** Functional annotation of the intersect file using ANNOVAR

Function	Percentage
Exonic	42.45
Intronic	50.74
Intergenic	0.04
Upstream/downstream	0.68
Untranslated region	4.96
Other	1.13
Exonic function	Percentage
Nonsynonymous	47.61
Synonymous	50.55
Stop gain/loss	0.50
Unknown	1.35

#### Variant identification

Of the 94,263 variants in the intersect file, 4425 variants were rare private variants and 8840 were known rare variants. In the analysis of 118 candidate genes, 807 variants were identified (Additional file 1: Table S1), and an additional 491 variants were identified in 134 genes located in regions ±25 kb of each known candidate gene (Additional file 1: Table S2). The summary of the annotation of these variants for all three categories are shown in Additional file 1: Table S3.

#### Family-based association and segregation analyses

The variants from each category were tested for association using a variance-component model for quantitative phenotypes (age at onset of cancer and age at onset of sarcoma) and disease status (cancer and sarcoma) in SOLAR. For those with no cancer onset, age at onset was set to 0. No variants were significantly associated with any of the four cancer outcomes after correcting for multiple testing. Any nominally associated variants (*p*-value < 0.05) were investigated for familial segregation in the three cancer cluster family pedigrees. No nominally significant associations were detected for Pedigree 1 after segregation analysis. Variants in three genes (*C16orf96, ABCB5,* and *PDIA2*) were detected in Pedigree 2 after segregation analysis. Variants in five genes (*ARHGAP39, ZFP69B, UVSSA, BEAN1,* and *KIF2C*) were nominally significant and segregated in Pedigree 3. A summary of these eight nominally associated variants are presented in Table 3.

**Table 3:** Summary of SOLAR association and segregation analysis by variant category

avSNP147	Chr^a^	Position	Gene	AA change/ Reg. Feature^b^	Family #	Cancer Outcome	p-value^c^	Q-value^d^	Variant Type	SIFT^e^	PolyPhen-2	Family MAF^f^	MAF 1 K ^g^
Rare Private Variants													
–	8	145,773,319	*ARHGAP39*	G1151A	3	Age at onset of cancer	0.01	1.00	NS	Deleterious	Deleterious	0.079	–
						Any Cancer	0.02	1.00					
Known Private Rare Variants													
rs191227556	16	4,606,552	*C16orf96*	T62C TF^h^ Binding + DNase Footprint + DNase Peak	2	Age at onset of cancer	0.01	1.00	NS/ REG	Deleterious	Deleterious	0.105	0.0002
						Any Cancer	0.01	1.00					
rs139741319	7	20,721,130	*ABCB5*	TF binding + matched TF motif + DNase peak	2	Age at onset of cancer	0.01	1.00	REG	–	–	0.105	0.0008
						Any Cancer	0.01	1.00					
rs139213019	1	40,929,077	*ZFP69B*	C1421G	3	Age at onset of cancer	0.01	1.00	NS	Deleterious	Deleterious	0.079	0.0016
						Any Cancer	0.02	1.00					
rs116741007	4	1,348,920	*UVSSA*	G1063A	3	Age at onset of cancer	0.01	1.00	NS	Deleterious	Deleterious	0.079	0..0040
						Any Cancer	0.02	1.00					
rs200706119	16	66,503,705	*BEAN1*	C226A	3	Age at onset of cancer	0.01	1.00	NS	Tolerated	–	0.079	0.0050
rs139373762	1	45,224,937	*KIF2C*	TF Binding + DNase Footprint + DNase Peak	3	Age at onset of cancer	0.01	1.00	REG	–	–	0.079	0.0012
Candidate Gene Variants													
rs45614840	16	334,543	*PDIA2*	C356G	2	Age at onset of cancer	0.01	1.00	NS	Deleterious	Deleterious	0.105	0.05
						Any Cancer	0.01	1.00					

^a^*Chr* Chromosome; ^b^Amino Acid Change or Regulatory Feature of the Observed Variants; ^c^Uncorrected p-value; ^d^Bonferroni corrected p-value for multiple testing (α = 0.05); ^e^*SIFT* Sorting Intolerant from Tolerant. ^f^Minor Allele Frequency in Family; ^g^MAF 1000G: Minor Allele Frequencies from the 1000 Genomes Database; ^h^*TF* Transcription Factor

### Validation

The eight candidate risk genes identified after family-based association and segregation analysis were subjected to variant burden analyses employing whole genome sequence data from 560 sarcoma cases from the ISKS and 1144 healthy ageing controls from the MGRB program accounting for European ancestry [19]. Information regarding the ISKS cases can be found in Additional file 1: Table S4. Of the 560 cases, the most common subtypes were sarcoma, not otherwise specified (15%), leiomyosarcoma, not otherwise specified (13%) and chondrosarcoma, not otherwise specified (7%). Of the 560 cases, 18 had secondary sarcoma, and one patient had four instances of sarcoma.

#### Variant burden analyses

One-sided Fisher’s exact tests were used to compare total burden of minor allele counts for rare deleterious variants (defined as deleterious in both SIFT and PolyPhen-2) and regulatory variants (defined as those with a RegulomeDB score < 3) to total major allele counts in cases and controls. The results of this analysis are shown in Table 4 (rare deleterious variants) and Table 5 (regulatory variants). None of the eight genes with rare deleterious variants were significant after correction for multiple testing but two genes (*ABCB5* and *C16orf96*) with regulatory variants showed significant association with burden testing after correction.

**Table 4:** Minor allele counts for rare nonsynonymous deleterious variants, odds ratios and *p*-values from Fisher’s exact test for genes of interest

Gene of interest	Allele Counts (# NS Variants) ISKS^a^	Allele Counts (# NS Variants) MGRB^b^	Odds ratio	*p*-value^c^	*Q-value* ^d^
*ABCB5*	30 (17)	32 (16)	1.79	0.02	0.16
*ARHGAP39*	1 (1)	3 (1)	2.07	0.45	1.00
*BEAN1*	1 (1)	0 (0)	0	1	1.00
*C16orf96*	4 (4)	12 (9)	1.81	0.296	0.237
*KIF2C*	0 (0)	1 (1)	0	1	1.00
*PDIA2*	5 (5)	1 (1)	4.07	0.23	1.00
*UVSSA*	21 (8)	25 (6)	1.29	0.45	1.00
*ZFP69B*	2 (2)	16 (4)	0.51	0.55	1.00

^a^*ISKS* International Sarcoma Kindred Study. ^b^*MGRB* Medical Genome Reference Bank. ^c^Uncorrected *p*-value. ^d^Bonferroni corrected *p*-value for multiple testing (α = 0.05)

**Table 5:** Minor allele counts for rare putative regulatory variants, odds ratios and *p*-values from Fisher’s exact test for genes of interest

Gene of interest	Allele Counts (# Reg. Variants) ISKS^a^	Allele Counts (#Reg. Variants) MGRB^b^	Odds ratio	*p*-value^c^	*Q-value* ^d^
*ABCB5*	12 (5)	3 (3)	4.9	0.007	0.049
*ARHGAP39*	10 (3)	24 (4)	1.13	0.702	1.00
*BEAN1*	3 (1)	1 (1)	6.11	0.10	0.800
*C16orf96*	58 (3)	151 (3)	0.58	0.0004	0.003
*KIF2C*	2 (1)	9 (1)	0.45	0.52	1.00
*PDIA2*	10 (1)	24 (1)	0.85	0.85	1.00
*UVSSA*	114 (4)	215 (4)	1.09	0.44	1.00
*ZFP69B*	0 (0)	0 (0)	–	–	–

^a^*ISKS* International Sarcoma Kindred Study. ^b^*MGRB* Medical Genome Reference Bank. ^c^Uncorrected *p*-value. ^d^Bonferroni corrected *p*-value for multiple testing (α = 0.05)

The *ABCB5* gene was found to have a nominally significantly higher burden (OR = 1.79, *p-value* = 0.02, q-value = 0.16, Table 4) associated with nonsynonymous deleterious variants and significant putative regulatory variants based on allele counts (OR = 4.9*, p*-value = 0.007, q-value = 0.049, Table 5) in sarcoma cases compared to controls. For deleterious variants within *ABCB5*, these gene burden association results are driven by higher heterozygosity at two variants (rs2074000, rs58795451) and lower heterozygosity at one variant (rs751879475). For the regulatory variants in *ABCB5*, this result is driven by higher heterozygosity at three variants (rs73684574, rs78879263, rs78155891). All three *ABCB5* variants have RegulomeDB scores of 2, suggesting that they have a likely impact on the transcription factor binding of this gene.

One other gene, *C16orf96,* was found to have a significantly lower burden (OR = 0.58*, p*-value = 0.0004, q-value = 0.003, Table 5) of regulatory variants in controls compared to sarcoma cases. This result was driven by the lower heterozygosity at two variants (rs11862083, rs76048912). The variant rs11862083 is a known eQTL with a RegulomeDB score of 1f and is linked to expression of *HSCARG* (also named NmrA-like family domain containing protein 1). The second variant, rs76048912, has a RegulomeDB score of 2 suggesting that this variant is likely to affect transcription factor binding.

## Discussion

An ever increasing number of genetic studies are utilizing genome-wide sequencing strategies in families to successfully identify novel susceptibility genes for human diseases. Recent examples include colorectal cancer, anaemia, Wilms tumour, prostate cancer, melanoma, and leukaemia, amongst others [33–41]. The two-stage study design typically used in these studies begin with WES of an initial small cohort of multi-case families ascertained from an affected proband that are negative for known causal mutations. Candidate variants are prioritized bioinformatically and by family-based segregation and association analysis followed by validation in a larger independent case and control cohort.

In this study our primary focus was sarcoma and we have used a two-stage study design to identify novel candidate susceptibility genes for sarcoma and other cancers. To the best of our knowledge this is the first study to successfully use this study design to identify novel risk genes for this rare group of cancers.

Three assumptions were made in determining familial segregation in this study. First, the variant will be rare (shared only by carriers in one family). Second, every carrier of a putative disease-causing variant will have the phenotype (complete penetrance). Third, every individual with the phenotype (cancer) will carry the putative disease-causing variant (100% probability of observing a genotype given the phenotype). These assumptions did not consider the possibility of unaffected carriers (incomplete penetrance), later onset of disease, or risk variants that occur in cases in more than one family. Therefore, some true variants may have been excluded using these strong assumptions.

Despite these limitations, by treating each cancer pedigree as a separate discovery unit we were able to identify novel rare variants showing nominal evidence of association with cancer risk in these families. Importantly, although not sufficient evidence on their own, these nominal variant associations pointed to candidate risk genes that we could then evaluate extensively in the second stage of our study design, dependent on the availability of large population cohorts of unrelated sarcoma cases and cancer free controls for which there was whole genome sequence; a powerful resource for gene validation.

Of the two novel candidate risk genes validated by variant burden analyses in stage two, the *C16orf96* open reading frame gene on chromosome 16 showed the strongest evidence of association with sarcoma risk. The function of this gene or any potential role for this gene in cancer pathogenesis has not been established. However, in silico analysis of regulatory elements associated with this gene demonstrate that it contains enhancers and promoters that target 11 genes, including Cell Death Inducing p53 Target protein 1 (*CDIP*), which is important for regulating TNF-alpha-mediated apoptosis in a p53/TP53-dependent manner [42]. In addition, one of the variants, rs11862083, driving this signal is known to be linked to the expression of the gene, *HSCARG*, which has been shown to be involved in histone H2A ubiquitination known to be involved in transcriptional repression and DNA damage response [43]. The *ABCB5* gene, although not previously associated with sarcoma, has been previously associated with clinical drug resistance and recurrence in malignant melanomas and leukaemias [44–48]. ABCB5-expressing cells have been shown to selectively survive when exposed to dacarbazine and other chemotherapeutic drugs [49].

## Conclusions

In this study we have provided evidence for two novel candidate risk genes for sarcoma. The two-stage genome-wide DNA sequencing study design we have employed is gaining momentum in the human disease genomics field as researchers return to family-based study designs to identify rare genetic variants now widely thought to account for some of the (substantial) missing heritability in complex diseases and traits. The current study adds to growing evidence that this approach, requiring only a relatively small number of affected families for initial gene discovery, can be successfully used to identify novel risk genes for complex human diseases, including rare cancers such as sarcoma. These novel risk genes will require functional evaluation in future studies. In addition, the clinical utility of these genes and associated variants in risk prediction models for relatives of cancer patients will also require further validation in other large independent studies, for example, the large Genomics England resource, that has a major focus on risk prediction for cancers.

## Additional file

Additional file 1: BMC Med Gen Submission - Jones 2019_Novel sarcoma risk genes in cancer cluster families_Supp Material. (DOCX 37 kb)

## Abbreviations

CDIP: Cell Death Inducing p53 Target protein 1
eQTL: Expression quantitative trait loci
ISKS: International Sarcoma Kindred Study
MAF: Minor allele frequency
MGRB: Medical Genome Reference Bank
OR: Odds Ratio
SIFT: Sorting Intolerant from Tolerant
SOLAR: Sequential Oligogenic Linkage Analysis Routines
TNF: Tumor necrosis factor
WES: Whole exome sequencing
