# Supplementary material for: Identification of novel sarcoma risk genes using a two-stage genome wide DNA sequencing strategy in cancer cluster families and population case and control cohorts
Source: BMC Med Genet. 2019 May 3;20:69. doi: 10.1186/s12881-019-0808-9 (PMC6499942; doi:10.1186/s12881-019-0808-9)
Supplement: Supplementary file 1 — BMC Med Gen Submission - Jones 2019_Novel sarcoma risk genes in cancer cluster families_Supp Material. (DOCX 37 kb) [file 12881_2019_808_MOESM1_ESM.docx]

# Additional file 1

**Information on the** International Sarcoma Kindred Study **an**d the Medical Genome Reference Bank

The International Sarcoma Kindred Study was initiated in 2008 to investigate the prevalence and nature of heritable risk in sarcoma populations (1). The International Sarcoma Kindred Study is a global genetic, biological, epidemiological, and clinical resource for researchers to investigate the hereditary characteristics of sarcoma. Patients were recruited from several sites across Australia, France, New Zealand, India, the United States of America and the United Kingdom.

Patients with sarcoma (probands) were recruited from major sarcoma treatment centers, regardless of their family history of cancer. Individuals with adult-onset sarcoma (> 15 years old) were eligible for the International Sarcoma Kindred Study. Family members were also invited to participate if the patient with sarcoma was < 45 years of age, or there was a significant family history of cancer (1). Study questionnaires containing demographic, medical, epidemiological and psychosocial information were completed, including personal history of cancer or exposure to known risk factors for sarcoma (2). Patients were also asked to donate a venous blood sample and tumor sample, as well as provide access to medical information and access to information about deceased relatives (collected from cancer registries and other health organizations). Medical history and treatment records were obtained for each proband where possible (2). All reported cancer diagnoses were independently verified by medical records, Australian and New Zealand cancer registries or death certificates.

The Medical Genome Reference Bank, funded by the NSW Office of Health and Medical Research, sequenced healthy, older individuals to create a high quality database depleted of damaging genetic variants (3). The Medical Genome Reference Bank program utilizes participants from two existing cohorts, the ASPirin in Reducing Events in the Elderly Study (4) and the 45 and Up study (5). The ASPirin in Reducing Events in the Elderly Study is an international clinical trial to determine whether daily low-dose aspirin improves the quality of life for 19,000 older people in Australia and the USA.^58^ No participants from the 45 and Up study were included in the current study.

Supplementary Table S1: 118 known cancer risk genes selected for candidate analysis

| **Gene name** | **Chromosome** | **Start^a^** | **End**^a^ | **Number of Variants** |
| --- | --- | --- | --- | --- |
| *APC* | 5 | 112018202 | 112206936 | 13 |
| *ARID1A* | 1 | 26997522 | 27133601 | 4 |
| *ATM* | 11 | 108068559 | 108264826 | 12 |
| *ATR* | 3 | 142143077 | 142322668 | 31 |
| *AXIN1* | 16 | 312440 | 427676 | 10 |
| *AXIN2* | 17 | 63499683 | 63582740 | 6 |
| *BARD1* | 2 | 215568275 | 215699428 | 10 |
| *BLM* | 15 | 91235579 | 91383686 | 9 |
| *BRCA1* | 17 | 41171312 | 41302500 | 13 |
| *BRCA2* | 13 | 32864617 | 32998809 | 20 |
| *BRIP1* | 17 | 59731547 | 59965920 | 9 |
| *BUB1B* | 2 | 111370409 | 111460684 | 1 |
| *C17orf85* | 17 | 3685045 | 3774545 | 3 |
| *CD99* | Y | 2534228 | 2634350 | 0 |
| *CDH1* | 16 | 68746195 | 68894444 | 6 |
| *CDKN2A* | 9 | 21942751 | 22000132 | 2 |
| *CHEK1* | 11 | 125471251 | 125552042 | 4 |
| *CHEK2* | 22 | 29058731 | 29162822 | 2 |
| *DDB2* | 11 | 47211493 | 47285769 | 4 |
| *DICER1* | 14 | 95527565 | 95633085 | 3 |
| *DKC1* | X | 153966031 | 154030964 | 0 |
| *DNA2* | 10 | 70148821 | 70256730 | 6 |
| *ELF3* | 1 | 201954690 | 202011315 | 3 |
| *ELF5* | 11 | 34475342 | 34560347 | 6 |
| *ERCC2* | 19 | 45829649 | 45898845 | 12 |
| *ERCC3* | 2 | 127989866 | 128076752 | 4 |
| *ERCC4* | 16 | 13989014 | 14071205 | 8 |
| *ERCC5* | 13 | 103479468 | 103549748 | 14 |
| *ERF* | 19 | 42726717 | 42784309 | 1 |
| *ERG* | 21 | 39726950 | 39895428 | 7 |
| *ETS1* | 11 | 128303656 | 128482453 | 4 |
| *ETS2* | 21 | 40152231 | 40221878 | 4 |
| *ETV-1* | 7 | 13905856 | 14054642 | 7 |
| *ETV2* | 19 | 36107647 | 36160773 | 4 |
| *ETV4* | 17 | 41580211 | 41648305 | 4 |
| *ETV6* | 12 | 11777788 | 12073325 | 2 |
| *EWSR1* | 22 | 29638998 | 29721515 | 8 |
| *EXT1* | 8 | 118786602 | 119149058 | 3 |
| *EXT2* | 11 | 44092747 | 44291980 | 6 |
| *FAM175A* | 4 | 84357094 | 84431290 | 3 |
| *FANCA* | 16 | 89778959 | 89908065 | 45 |
| *FANCB* | X | 14836529 | 14916184 | 0 |
| *FANCC* | 9 | 97836336 | 98104991 | 3 |
| *FANCD2* | 3 | 10043113 | 10166344 | 12 |
| *FANCE* | 6 | 35395138 | 35459881 | 7 |
| *FANCF* | 11 | 22619079 | 22672387 | 2 |
| *FANCG* | 9 | 35048835 | 35105013 | 3 |
| *FANCI* | 15 | 89762194 | 89885362 | 13 |
| *FANCL* | 2 | 58361378 | 58493515 | 4 |
| *FANCM* | 14 | 45580136 | 45695093 | 9 |
| *FH* | 1 | 241635857 | 241708085 | 3 |
| *FLI1* | 11 | 128538811 | 128708162 | 3 |
| *HNF4A* | 20 | 42959441 | 43061115 | 7 |
| *IDH1* | 2 | 209075953 | 209144806 | 3 |
| *IDH2* | 15 | 90602212 | 90670708 | 4 |
| *KIF1B* | 1 | 10245764 | 10466661 | 27 |
| *KIT* | 4 | 55499095 | 55631881 | 4 |
| *LIG1* | 19 | 48593703 | 48698560 | 24 |
| *LIG4* | 13 | 108834792 | 108892882 | 2 |
| *MDM2* | 12 | 69176971 | 69264320 | 4 |
| *MEN1* | 11 | 64545986 | 64603188 | 8 |
| *MET* | 7 | 116287459 | 116463440 | 7 |
| *MLH1* | 3 | 37009841 | 37117337 | 6 |
| *MLH3* | 14 | 75455467 | 75543235 | 8 |
| *MRE11A* | 11 | 94125469 | 94252040 | 5 |
| *MSH2* | 2 | 47605206 | 47735367 | 11 |
| *MSH3* | 5 | 79925467 | 80197634 | 13 |
| *MSH6* | 2 | 47985221 | 48059092 | 11 |
| *MUTYH* | 1 | 45769914 | 45831142 | 6 |
| *NBN* | 8 | 90920564 | 91021899 | 10 |
| *NEIL2* | 8 | 11602172 | 11669854 | 5 |
| *NF1* | 17 | 29396945 | 29729695 | 8 |
| *NF2* | 22 | 29974545 | 30119589 | 2 |
| *PALB2* | 16 | 23589483 | 23677678 | 3 |
| *PMS1* | 2 | 190623811 | 190767355 | 3 |
| *PMS2* | 7 | 5987870 | 6073737 | 5 |
| *POLH* | 6 | 43518878 | 43613260 | 4 |
| *PPARG* | 3 | 12368001 | 12500855 | 3 |
| *PRKAR1A* | 17 | 66482921 | 66554570 | 6 |
| *PTCH1* | 9 | 98180264 | 98295831 | 9 |
| *PTEN* | 10 | 89598195 | 89753532 | 2 |
| *PTPN11* | 12 | 112831536 | 112972717 | 2 |
| *RAD50* | 5 | 131867616 | 132005313 | 6 |
| *RAD51C* | 17 | 56744963 | 56836692 | 2 |
| *RAD51D* | 17 | 33401811 | 33458500 | 3 |
| *RB1* | 13 | 48852883 | 49081026 | 9 |
| *RECQL4* | 8 | 145711667 | 145768210 | 10 |
| *RET* | 10 | 43547517 | 43650797 | 12 |
| *RMI1* | 9 | 86570637 | 86643987 | 2 |
| *RMI2* | 16 | 11414311 | 11470617 | 8 |
| *RPA1* | 17 | 1708273 | 1827848 | 8 |
| *RPA3* | 7 | 7651575 | 7783238 | 1 |
| *RPS19* | 19 | 42338988 | 42400484 | 3 |
| *SDHA* | 5 | 193356 | 281814 | 12 |
| *SDHB* | 1 | 17320225 | 17405665 | 3 |
| *SDHC* | 1 | 161259166 | 161359535 | 3 |
| *SDHD* | 11 | 111932548 | 111991525 | 3 |
| *SMARCA4* | 19 | 11046598 | 11197958 | 11 |
| *SMARCB1* | 22 | 24104150 | 24201705 | 3 |
| *SPDEF* | 6 | 34480579 | 34549110 | 1 |
| *SPI1* | 11 | 47351409 | 47425127 | 4 |
| *SQSTM1* | 5 | 179222842 | 179290077 | 3 |
| *STK11* | 19 | 1180798 | 1253434 | 7 |
| *TAF15* | 17 | 34111459 | 34199246 | 4 |
| *TGFBR2* | 3 | 30622994 | 30760633 | 2 |
| *TNFRSF11A* | 18 | 59967520 | 60079943 | 8 |
| *TOP1* | 20 | 39632462 | 39778126 | 0 |
| *TOP3A* | 17 | 18152235 | 18243321 | 8 |
| *TP53* | 17 | 7546720 | 7615868 | 5 |
| *TP53BP1* | 15 | 43674412 | 43810354 | 5 |
| *TSC1* | 9 | 135741735 | 135845020 | 2 |
| *TSC2* | 16 | 2072990 | 2163713 | 22 |
| *VHL* | 3 | 10158319 | 10220354 | 1 |
| *WRN* | 8 | 30865778 | 31056277 | 25 |
| *WT1* | 11 | 32384322 | 32482081 | 9 |
| *XPA* | 9 | 100412191 | 100484691 | 2 |
| *XPC* | 3 | 14161648 | 14245172 | 14 |
| *XRCC2* | 7 | 152318587 | 152398250 | 2 |

^a^ Start and End: the chromosome locations of the start and end of the gene (including ± 25 kb)

Supplementary Table S2: Genes harbouring variants ±25 kb of the 118 known cancer genes

| Gene | Chromosome | Number of variants |
| --- | --- | --- |
| *ACCS* | 11 | 8 |
| *ACP2* | 11 | 4 |
| *ACRV1* | 11 | 1 |
| *ACYP1* | 14 | 1 |
| *AIMP2* | 7 | 3 |
| *ALX4* | 11 | 2 |
| *ANKRD49* | 11 | 1 |
| *ARHGAP39* | 8 | 5 |
| *ARHGDIG* | 16 | 2 |
| *ARHGEF1* | 19 | 2 |
| *ATP13A2* | 1 | 4 |
| *ATP1B2* | 17 | 3 |
| *ATP5D* | 19 | 3 |
| *BRK1* | 3 | 3 |
| *C11orf57* | 11 | 2 |
| *C11orf97* | 11 | 3 |
| *C19orf26* | 19 | 5 |
| *C22orf15* | 22 | 2 |
| *C5orf45* | 5 | 7 |
| *C9orf9* | 9 | 1 |
| *CAMKK1* | 17 | 5 |
| *CAT* | 11 | 2 |
| *CCDC127* | 5 | 2 |
| *CDC42BPG* | 11 | 12 |
| *CFAP126* | 1 | 3 |
| *CHCHD10* | 22 | 2 |
| *COX6B1* | 19 | 2 |
| *CPM* | 12 | 4 |
| *DCTN5* | 16 | 5 |
| *DERL3* | 22 | 4 |
| *DHFR* | 5 | 3 |
| *DHX8* | 17 | 5 |
| *DLAT* | 11 | 1 |
| *DMRTC2* | 19 | 1 |
| *EIF2AK1* | 7 | 1 |
| *EIF2B2* | 14 | 2 |
| *EPCAM* | 2 | 2 |
| *EPM2AIP1* | 3 | 1 |
| *EVI2A* | 17 | 2 |
| *FAM20A* | 17 | 3 |
| *FAM20A,PRKAR1A* | 17 | 1 |
| *FBXO11* | 2 | 1 |
| *FDFT1* | 8 | 4 |
| *FLII* | 17 | 7 |
| *FNDC8* | 17 | 2 |
| *FRY* | 13 | 1 |
| *GAS2L1* | 22 | 5 |
| *GATA4* | 8 | 3 |
| *GPT* | 8 | 2 |
| *GSK3A* | 19 | 3 |
| *GTPBP2* | 6 | 2 |
| *HAUS5* | 19 | 3 |
| *HEATR9* | 17 | 1 |
| *HELQ* | 4 | 11 |
| *HNRNPK* | 9 | 4 |
| *HSCB* | 22 | 2 |
| *IL13* | 5 | 2 |
| *INTS2* | 17 | 2 |
| *IRAK2* | 3 | 4 |
| *ITFG3* | 16 | 2 |
| *ITGAE* | 17 | 1 |
| *KLC3* | 19 | 8 |
| *LOC100507346* | 9 | 3 |
| *LOC401052* | 3 | 4 |
| *LPAR6* | 13 | 1 |
| *LRRC14* | 8 | 3 |
| *LRRC14B* | 5 | 2 |
| *LRRFIP2* | 3 | 2 |
| *LYPD4* | 19 | 3 |
| *MAD2L1BP* | 6 | 1 |
| *MAP3K2* | 2 | 1 |
| *MAP4K2* | 11 | 5 |
| *MFSD3* | 8 | 1 |
| *MIDN* | 19 | 3 |
| *MIEF2* | 17 | 9 |
| *MIS18BP1* | 14 | 2 |
| *MMP11* | 22 | 4 |
| *MPZ* | 1 | 1 |
| *MRPL28* | 16 | 11 |
| *MRPS18C* | 4 | 1 |
| *MYBPC3* | 11 | 13 |
| *NDUFAB1* | 16 | 2 |
| *NPAT* | 11 | 1 |
| *NR1H3* | 11 | 2 |
| *ORMDL1* | 2 | 3 |
| *OSGIN2* | 8 | 2 |
| *PACSIN1* | 6 | 1 |
| *PADI2* | 1 | 9 |
| *PDIA2* | 16 | 10 |
| *PGD* | 1 | 3 |
| *PIGO* | 9 | 10 |
| *PIGV* | 1 | 2 |
| *PIH1D2* | 11 | 1 |
| *PKD1* | 16 | 17 |
| *PLA2G4C* | 19 | 10 |
| *PLCG1-AS1* | 19 | 1 |
| *POLG* | 15 | 11 |
| *PPP1R16A* | 8 | 2 |
| *R3HDML* | 20 | 6 |
| *RBM42* | 19 | 4 |
| *RCBTB2* | 13 | 1 |
| *RGS11* | 16 | 5 |
| *RHBDD3* | 22 | 2 |
| *RNPEP* | 1 | 5 |
| *RNU6-28P* | 15 | 5 |
| *RPL10A* | 6 | 1 |
| *RUFY2* | 10 | 2 |
| *SHMT1* | 17 | 5 |
| *SLC2A11* | 22 | 1 |
| *SLC9A3R2* | 16 | 6 |
| *SMCR8* | 17 | 6 |
| *SMYD4* | 17 | 3 |
| *SRP19* | 5 | 2 |
| *STOML2* | 9 | 3 |
| *STT3A* | 11 | 3 |
| *TANGO6* | 16 | 2 |
| *TEAD3* | 6 | 4 |
| *TESK2* | 1 | 3 |
| *TMEM43* | 3 | 10 |
| *TMEM8A* | 16 | 13 |
| *TOE1* | 1 | 1 |
| *UPK1A* | 19 | 3 |
| *VAT1* | 17 | 1 |
| *VCP* | 9 | 5 |
| *VPS9D1* | 16 | 2 |
| *VRK2* | 2 | 1 |
| *WRAP53* | 17 | 5 |
| *XPO5* | 6 | 2 |
| *XRN1* | 3 | 1 |
| *ZAR1L* | 13 | 4 |
| *ZC2HC1C* | 14 | 2 |
| *ZNF276* | 16 | 13 |
| *ZNF526* | 19 | 1 |
| *ZNF710* | 15 | 6 |

Supplementary Table S3: Summary of variant annotation using ANNOVAR and RegulomeDB

| Categories | Rare private | Known rare | Candidate gene |
| --- | --- | --- | --- |
| Number of variants | 4,425 | 8,840 | 1,297 |
| Location |  |  |  |
| Exonic | 1,858 | 5,184 | 487 |
| Intronic | 2,170 | 3,209 | 724 |
| Downstream | 8 | 6 | 5 |
| Upstream | 25 | 14 | 3 |
| 5’ untranslated region | 132 | 124 | 28 |
| 3’ untranslated region | 119 | 197 | 38 |
| Splicing | 19 | 19 | 2 |
| Non-coding RNA | 91 | 84 | 10 |
| Intergenic | 1 | 3 | 0 |
| Upstream/downstream | 1 | 0 | 0 |
| 5’/3’ untranslated region | 1 | 0 | 0 |
| Exonic function |  |  |  |
| Nonsynonymous | 1,184 | 2,815 | 211 |
| Stop gain | 40 | 34 | 1 |
| Stop loss | 1 | 4 | 0 |
| Synonymous | 601 | 2,268 | 273 |
| Unknown | 32 | 63 | 2 |
| Functional prediction |  |  |  |
| Deleterious in SIFT and PolyPhen-2 | 254 | 449 | 22 |
| Tolerated in SIFT and PolyPhen-2 | 545 | 1,551 | 134 |
| Unknown in SIFT and PolyPhen-2 | 3,189 | 46 | 6 |
| Regulome database score < 3 | 0 | 683 | 168 |

Supplementary Table S4: Morphology of cases from the International Sarcoma Kindred Cohort

| **Morphology** | **Count** |
| --- | --- |
| Abdominal fibromatosis | 1 |
| Adenosarcoma | 1 |
| Alveolar rhabdomyosarcoma | 6 |
| Alveolar soft part sarcoma | 3 |
| Angiolipoma, NOS | 1 |
| Angiomatoid fibrous histiocytoma | 1 |
| Angiomyosarcoma | 4 |
| Angiosarcoma | 9 |
| Carcinosarcoma, NOS | 1 |
| Chondroblastic osteosarcoma | 4 |
| Chondrosarcoma, NOS | 40 |
| Chordoma, NOS | 2 |
| Clear cell chondrosarcoma | 2 |
| Clear cell sarcoma, NOS | 3 |
| Dedifferentiated chondrosarcoma | 3 |
| Dedifferentiated liposarcoma | 6 |
| Dermatofibrosarcoma protuberans, NOS | 6 |
| Dermatofibrosarcoma, NOS | 3 |
| Desmoplastic small round cell tumor | 2 |
| Embryonal rhabdomyosarcoma, NOS | 2 |
| Endometrial stromal sarcoma, low grade | 3 |
| Endometrial stromal sarcoma, NOS | 3 |
| Epithelioid hemangioendothelioma, malignant | 4 |
| Epithelioid sarcoma | 6 |
| Ewing sarcoma | 33 |
| Fibromyxosarcoma | 5 |
| Fibrosarcoma, NOS | 2 |
| Gastrointestinal stromal sarcoma | 22 |
| Gastrointestinal stromal tumor, NOS | 13 |
| Giant cell sarcoma | 1 |
| Giant cell tumor of bone, malignant | 1 |
| Hemangioendothelioma, malignant | 1 |
| Hemangiopericytoma, malignant | 2 |
| Hemangiopericytoma, NOS | 2 |
| Histiocytic sarcoma | 1 |
| Infantile fibrosarcoma | 1 |
| Leiomyosarcoma, NOS | 75 |
| Liposarcoma, NOS | 16 |
| Liposarcoma, well differentiated | 24 |
| Malignant fibrous histiocytoma | 14 |
| Malignant peripheral nerve sheath tumor | 7 |
| Malignant peripheral nerve sheath tumor with rhabdomyoblastic differentiation | 1 |
| Malignant rhabdoid tumor | 1 |
| Meningioma, NOS | 1 |
| Myxoid chondrosarcoma | 4 |
| Myxoid liposarcoma | 22 |
| Myxopapillary ependymoma | 1 |
| Ossifying fibromyxoid tumor | 1 |
| Osteochondroma | 1 |
| Osteosarcoma in Paget disease of bone | 1 |
| Osteosarcoma, NOS | 31 |
| Paraganglioma, NOS | 1 |
| Parosteal osteosarcoma | 3 |
| Periosteal osteosarcoma | 1 |
| Peripheral neuroectodermal tumor | 2 |
| Phyllodes tumor, malignant | 2 |
| Pleomorphic liposarcoma | 4 |
| Primitive neuroectodermal tumor, NOS | 4 |
| Rhabdomyosarcoma, NOS | 3 |
| Round cell liposarcoma | 1 |
| Sarcoma, NOS | 84 |
| Small cell sarcoma | 1 |
| Solitary fibrous tumor | 3 |
| Solitary fibrous tumor, malignant | 2 |
| Spindle cell sarcoma | 8 |
| Stromal sarcoma, NOS | 2 |
| Synovial sarcoma, biphasic | 10 |
| Synovial sarcoma, epithelioid cell | 1 |
| Synovial sarcoma, monophasic fibrous | 4 |
| Synovial sarcoma, NOS | 19 |
| Synovial sarcoma, spindle cell | 2 |
| Telangiectatic osteosarcoma | 1 |
| Undifferentiated sarcoma | 2 |

NOS: not otherwise specified.

# References

1. Australasian Sarcoma Study Group. International Sarcoma Kindred Study Melbourne: Australasian Sarcoma Study Group; 2013 [Available from: <http://www.australiansarcomagroup.org/sarcomakindredstudy>.

2. Mitchell G, Ballinger ML, Wong S, Hewitt C, James P, Young M-A, et al. High frequency of germline TP53 mutations in a prospective adult-onset sarcoma cohort. PLOS ONE. 2013;8(7):1-7.

3. Garvan Institute of Medical Research. Medical genome reference bank Garvan Institute of Medical Research2017 [Available from: <https://www.garvan.org.au/research/kinghorn-centre-for-clinical-genomics/clinical-genomics/sydney-genomics-collaborative/mgrb>.

4. McNeil JJ, Woods RL, Nelson MR, Murray AM, Reid CM, Kirpach B, et al. Baseline characteristics of participants in the ASPREE (ASPirin in Reducing Events in the Elderly) study. The Journals of Gerontology. 2017.

5. 45 and Up Study Collaborators. Cohort Profile: the 45 and Up Study. International Journal of Epidemiology. 2008;37(5):941-7.
